# Characterization of laser powder bed fusion metal samples using Bragg edge neutron transmission analysis

**DOI:** 10.1107/S1600576726001482

**Published:** 2026-03-31

**Authors:** Matilde Dematteis, Luisa Vigorelli, Francesco Grazzi, Donato Orlandi, Daniele Cortis, Matteo Busi, Marco Costa

**Affiliations:** a Dipartimento di Fisica Università degli Studi di Torino and INFN (TO) Torino 10125 Italy; b Dipartimento di Fisica ‘Giuseppe Occhialini’, Università degli Studi di Milano–Bicocca and INFN (MI), Milano, 20126, Italy; c Consiglio Nazionale delle Ricerche Istituto di Fisica Applicata `Nello Carrara' and INFN (FI) Sesto Fiorentino (FI) 50019 Italy; d Laboratori Nazionali del Gran Sasso, INFN Assergi (AQ) 67100 Italy; e PSI Center for Neutron and Muon Sciences, Villigen PSI, 5232, Switzerland; Technical University of Denmark, Denmark

**Keywords:** neutron Bragg edge imaging, residual strain, texture, additive manufacturing

## Abstract

Bragg edge neutron transmission analysis is a non-destructive technique that can be used for the investigation of properties of crystalline solids, such as microstructure, texture, strain or defects. In this work, Bragg edge imaging is applied to characterize additively manufactured metal samples produced via powder bed fusion–laser-based, featuring an innovative star-shaped geometry. This process can induce microstructural inhomogeneities within the material, thereby compromising the mechanical integrity of the final component. For this reason, a comprehensive understanding of the manufacturing process is essential to identify optimal operational parameters. Because of the lack of non-invasive techniques allowing an in-depth study of the microstructure of these samples, Bragg edge imaging is applied for providing detailed quantitative information on the manufacturing process. In this context, the final aim of this work is to investigate how the production process influences the final manufactured components. To study these effects, three different additively manufactured samples made of different metal alloys have been characterized by Bragg edge analysis. Characterization of elastic lattice strain, density of crystallographic defects and texture reveals significant discrepancies between the samples and their respective starting powders. These findings elucidate the various effects induced by the manufacturing process, which alters the crystalline structure of the metal and introduces anisotropy, potentially leading to mechanical failure of the components.

## Abstract 2

Additive manufacturing represents a highly promising technique across various research and development sectors. In this work, Bragg edge neutron imaging is employed to characterize the crystallographic structure of additively manufactured metal samples, aiming to validate and optimize the production process.

## 1. Introduction

Over the past few decades, additive manufacturing (AM) has emerged as a key technology for producing 3D components through layer-by-layer addition of material (Gibson *et al.*, 2021 ▸). This process allows components to be built with complex geometries that could not be produced by traditional techniques (*e.g.* cutting/milling). It can be used with various materials (polymers, metals) to create parts for prototyping, tooling and end-use products, enabling faster innovation, less waste and greater design freedom. Among the different methods, powder bed fusion–laser-based (PBF-LB) is particularly employed for the production of metallic parts, using the thermal energy of a laser to selectively melt the metal powder in a powder bed (Yadroitsev *et al.*, 2021 ▸; Yap *et al.*, 2015 ▸). After the production, each workpiece can be subjected to post-production analysis and treatments such as relative density measurements, metallographic analyses and thermal relaxation to test the quality of the manufacturing process (Kantaros *et al.*, 2024 ▸; Peng *et al.*, 2021 ▸). In addition, further non-invasive methods can be employed for final characterization, such as X-ray tomography for inner volume inspection (Withers *et al.*, 2021 ▸; Zanini *et al.*, 2018 ▸). However, due to the high X-ray attenuation power of high-*Z* materials, this kind of analysis performed on metal samples is difficult and poorly sensitive to metal microstructures. Therefore, neutron-based techniques (*e.g.* imaging and diffraction methods) represent a valid alternative to X-ray analysis: thanks to their high penetration power in the bulk of metallic components, they allow detailed study of the crystalline structure (Langel, 2023 ▸).

Neutron diffraction is an established and powerful method for studying the crystal structure of materials (Withers, 2007 ▸; Woo *et al.*, 2011 ▸). Due to their wavelength, comparable to interatomic spacing, neutrons undergo elastic scattering within the lattice. The resulting diffraction pattern allows the determination of lattice parameter variations, providing a fundamental basis for the quantitative analysis of internal strain distributions within the material bulk. On the other hand, neutron imaging modalities based on diffraction contrast enable one to obtain information on local density, strains, phase composition and texture variations (Woracek *et al.*, 2018 ▸; Santisteban *et al.*, 2002 ▸). While diffraction techniques provide structural insights, imaging methods enable the spatial mapping of the investigated parameters, revealing their distribution across the entire sample. In this study we employ the wavelength-dispersive neutron imaging technique called Bragg edge neutron transmission (BENT) (Vogel, 2000 ▸), exploited in two (projection) or three (tomography) dimensions; it can also be used to perform time-resolved studies (Watkins *et al.*, 2013 ▸; Wensrich *et al.*, 2016 ▸; Woracek *et al.*, 2014 ▸; Woracek *et al.*, 2015 ▸). It is demonstrated that BENT imaging can be applied efficiently to assess the residual stress induced in additively manufactured samples, allowing the effects of the manufacturing itself and post-processing treatments to be analyzed (Busi *et al.*, 2021 ▸; Ramadhan *et al.*, 2022 ▸; Su *et al.*, 2021 ▸).

In this work, three samples realized with the PBF-LB technique using different metal alloys (stainless steel 316L, low-carbon steel 16MnCr5 and pure copper) have been produced at the Laboratori Nazionali del Gran Sasso of the Istituto Nazionale di Fisica Nucleare (LNGS-INFN). They have been studied by means of Bragg edge imaging at BOA (beamline for neutron optics and other approaches) at the Paul Scherrer Institute (PSI) in Switzerland, in order to characterize them in terms of defects and microstructure changes introduced by the manufacturing process. The use of BENT as a first-step investigation method allows for 2D sample mapping, representing an interesting expansion of this technique in the AM field. Following this preliminary characterization, neutron diffraction and neutron tomography will be performed to obtain complementary information and deeper insights into the crystallographic structure of the samples. Although preliminary, the results of this analysis will provide crucial information about the optimization of production conditions, one of the final goals of the project.

The paper is organized as follows: Section 2 details the experimental procedure, including sample preparation and instrumental setup. The core analysis is described in Section 3, which covers the experimental results and the discussion. Finally, Section 4 summarizes the main findings and provides future perspectives.

## 2. Experiment

### 2.1. Characterization method

Neutron Bragg edge imaging, used to measure the samples in this work, provides transmission spectra for all pixels of the detector by recording a series of sample images with different incident wavelengths. By studying the entire transmission spectrum, information on the crystalline structure of the sample can be obtained from the evaluation of 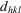, the distance between lattice planes of the family {*hkl*} (Grazzi *et al.*, 2021 ▸). According to the Bragg law
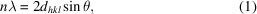
this distance is related to the wavelength λ of the incident radiation. Therefore, to investigate the crystalline structure, it is necessary to perform a wavelength scan and measure the transmitted intensity for each selected λ.

**Figure 1:**
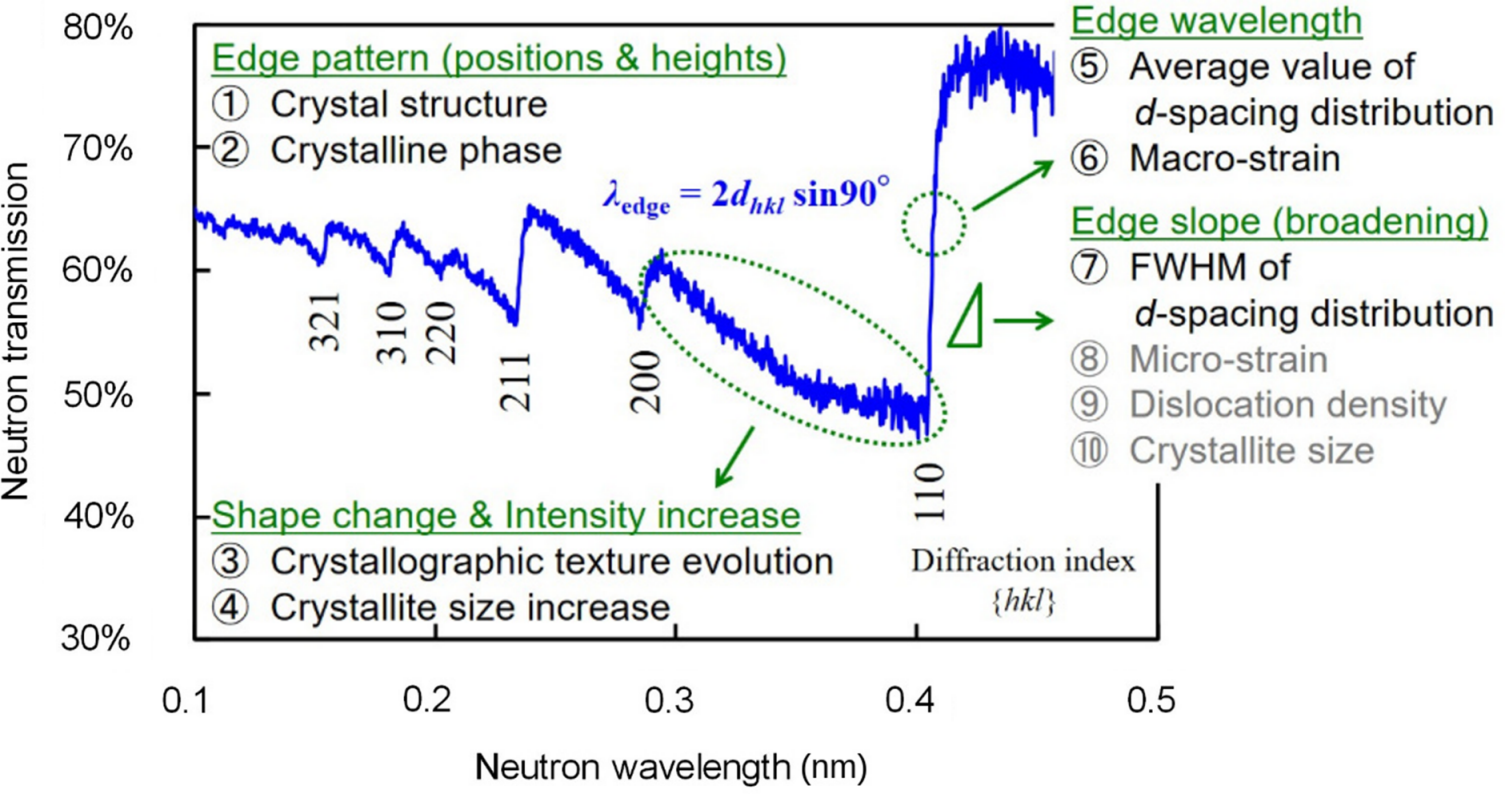
Bragg edge transmission spectrum of iron as a function of neutron wavelength, and crystalline structural information that can be obtained from the spectrum (Sato, 2018 ▸).

From equation (1) it follows that no radiation of a wavelength larger than 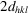 can be diffracted by a particular set of lattice planes {*hkl*}. This condition results in a sudden and well defined increase of the transmitted intensity at 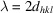. This sharp rise is called the Bragg edge (Sato *et al.*, 2013 ▸). In Fig. 1 ▸ a transmission spectrum is shown: the Bragg pattern is clearly visible and for each Bragg edge the corresponding {*hkl*} Miller indices are indicated. The main parameters that describe such a spectrum are the position, height and width of the Bragg edge. The wavelength of the edge position is directly related to the interplanar distance 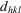, giving information on the *d*-spacing distribution through the sample and allowing one to study the strain distribution inside the object. The height of the Bragg edge is instead related to the crystalline phase of the material and to the presence of preferred orientations among the grains. The edge width, together with the *d* spacing, is related to the density of crystallographic defects (Sato, 2018 ▸).

### 2.2. Sample geometry and production process

**Figure 2:**
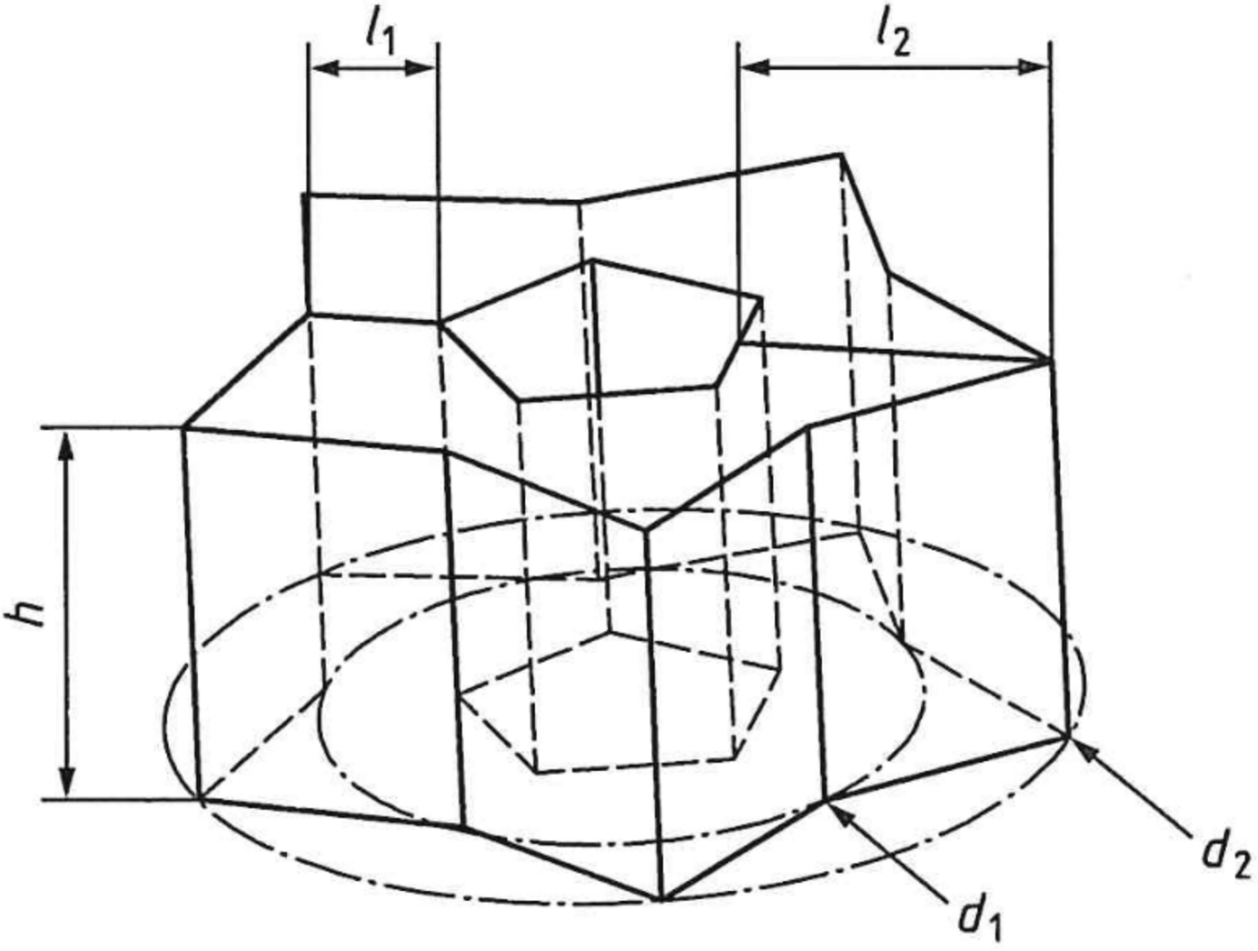
General sketch showing the main dimensions of the sample geometry (ISO, 2023 ▸).

PBF-LB technology has been used to produce the samples to be analyzed by BENT. This technology allows the production of complex metal parts layer by layer using a highly focused laser source to melt a fine layer of powder. The process involves the spreading of the powder on a substrate (*i.e.* building platform), the laser scanning of the cross sections of geometry, the lowering of the building platform and repetition of the process until the object is complete. In this case, three different metals have been manufactured: (i) 316L stainless steel, (ii) 16MnCr5 low-carbon steel and (iii) pure Cu. Different materials were selected to evaluate the performance of the machine through different manufacturing conditions. The sample geometry (Fig. 2 ▸) has been defined using as reference the ISO ASTM TR 52905 Standard (ISO, 2023 ▸), which describes and proposes specific configurations and shapes for applications in non-destructive testing. The main dimensions, based on material density (ρ), are reported in Table 1 ▸, where *h* is the height, 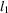 the thin-wall section, 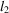 the thick-wall section, and 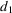 the inner and 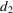 the outer star diameter. To ensure sufficient intensity of the transmitted neutron beam during the measurements, the sample height was reduced from the 45 mm specified in the standard to 18 mm.

The production was carried out at LNGS-INFN (Orlandi & Cortis, 2023 ▸) by means of a SISMA MySint100 PM-RM machine equipped with a laser source of 200 W, a fixed laser spot of 30 µm and a cylindrical building platform (100 mm diameter and 100 mm height). The metal powders were provided by Metals4Printing company with an average grain size in the range of 15–45 µm. The PBF-LB process parameters, such as laser power (*P*), scanning speed (*S*), hatch distance (*H*) and layer thickness (*L*) were previously optimized and validated (Cortis *et al.*, 2024 ▸; Cortis *et al.*, 2025 ▸). Table 2 ▸ reports the process parameters for each material together with the volumetric energy density (VED) applied by the laser source on the powder bed:
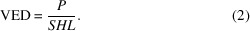
VED can be considered a descriptive variable of the set of parameters in order to compare and evaluate the PBF-LB process. The relative density (ρ) for each material was evaluated using the Archimedes’ principle. The low density of Cu (≃ 90%), with respect to other steel alloys (>99%), is attributed to the low absorbance and high reflectivity of this material (Alphonso *et al.*, 2023 ▸; Hummel *et al.*, 2021 ▸) at the infrared wavelengths of the PBF-LB laser machine (*i.e.* 1070 nm). The manufacturing process took place under argon gas atmosphere (*i.e.* oxygen level <0.1%) with a constant flow rate of 2.5 m s^−1^. The same Meander scanning strategy (Duong *et al.*, 2022 ▸; Yan *et al.*, 2019 ▸) was employed for all samples. This strategy involves the moving of the laser path back and forth within a layer in order to obtain a better heat balance than can be achieved with simple unidirectional scans. However, this solution can produce a high thermal gradient and columnar grains due to localized heating and directional growth. To overcome this aspect, a 67° rotation after each layer has been imposed. The rotation between consecutive layers helps to randomize grain growth, producing a more isotropic microstructure, reducing residual stresses and improving mechanical properties by avoiding the columnar solidification patterns. Finally, samples were produced with the height parallel to the building direction (*z* axis) and with support structures only on the bottom surfaces.

**Figure 3:**
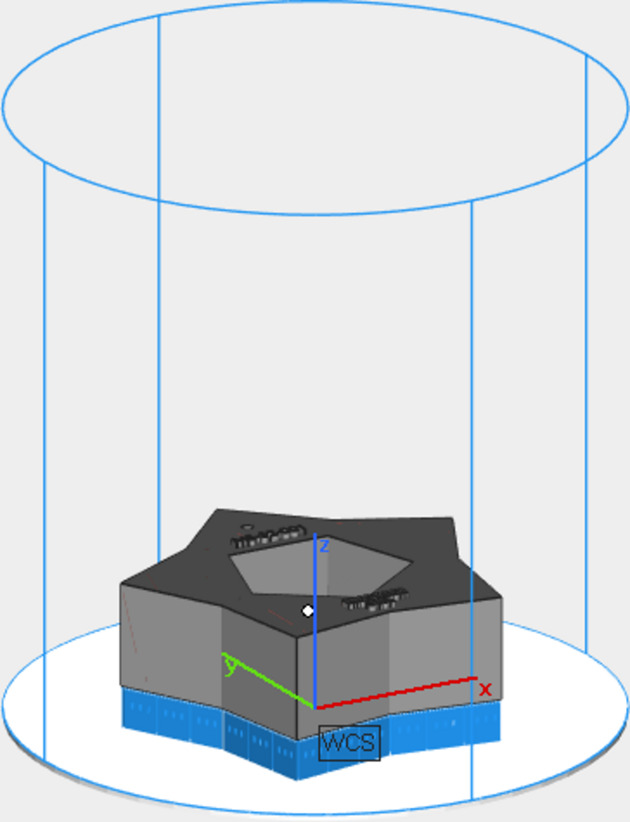
Example of the sample inside the envelope of the building platform.

**Figure 4:**
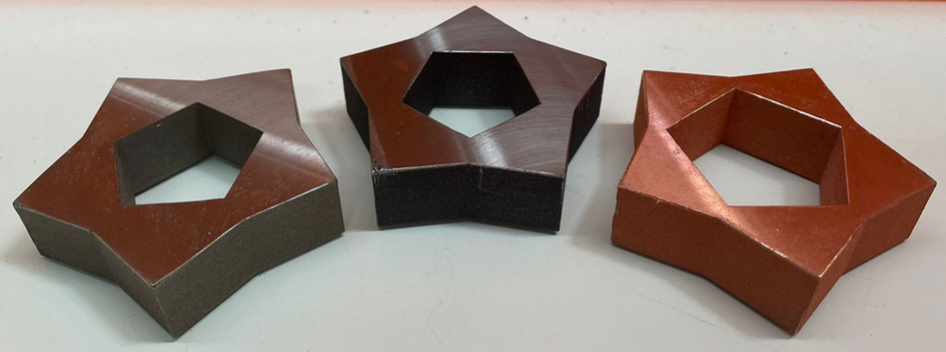
Samples manufactured using PBF-LB at the AM laboratory of the Laboratori Nazionali del Gran Sasso. Left: 316L stainless steel. Center: 16MnCr5 low-carbon steel. Right: copper.

Fig. 3 ▸ shows an example of the sample inside the envelope of the building platform of the PBF-LB machine. The blue parts are the solid structures, directly in contact with the substrate, while the gray part is the sample being built. The *y* axis is the direction of the gas flow, while the *x* axis is the direction of distribution of the metal powder. After the production, samples (Fig. 4 ▸) were cut from the building platform and post-processed with surface sandblasting (Teo *et al.*, 2021 ▸).

### 2.3. Experimental setup

**Figure 5:**
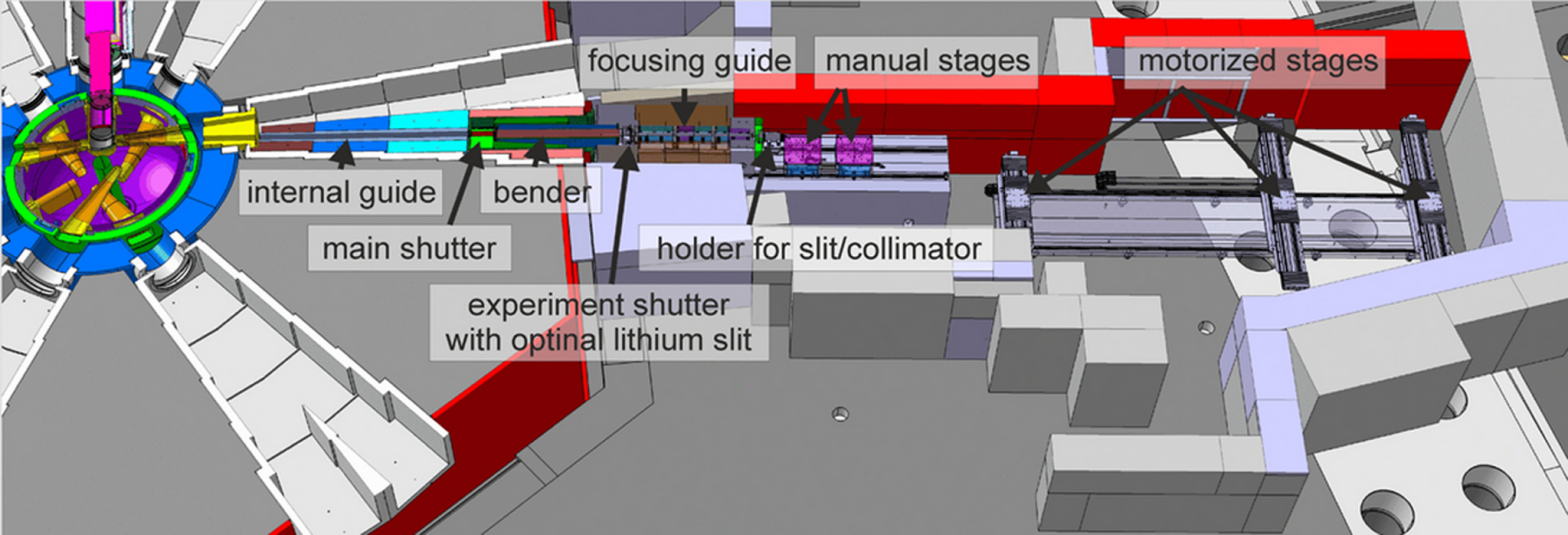
Layout of the BOA beamline at the SINQ neutron source (Morgano *et al.*, 2014 ▸).

The samples were measured at BOA, a neutron beamline on SINQ (Bauer, 1998 ▸), the spallation neutron source of the PSI. Fig. 5 ▸ outlines the layout of BOA. This facility is a cold neutron beamline with a wavelength range from 1 to 20 Å (Morgano *et al.*, 2014 ▸).

#### 2.3.1. Double-crystal monochromator

In order to select a particular energy band out of a white neutron beam, for this data acquisition a double-crystal monochromator (DCM) was used; this device is able to select only a narrow energy band from a white beam by using two crystals to reflect, through Bragg scattering, only one particular wavelength. By rotating and shifting the crystals, according to equation (1) it is possible to select the energy of the outgoing neutrons (Morgano *et al.*, 2014 ▸).

#### 2.3.2. Detector system

The detector system used for this work is a scintillator and CCD-camera-based system. A Cu-doped ^6^LiF(ZnS) scintillator was used, where the lithium fluoride is added to the zinc sulfide because of its extremely high thermal neutron cross section (940 barn) (Korotcenkov & Ivanov, 2023 ▸). The choice of this specific scintillator derives from the fact that it generates scintillation light with green wavelength, the one that maximizes the efficiency of the CCD camera used.

#### 2.3.3. Sample disposition

**Figure 6:**
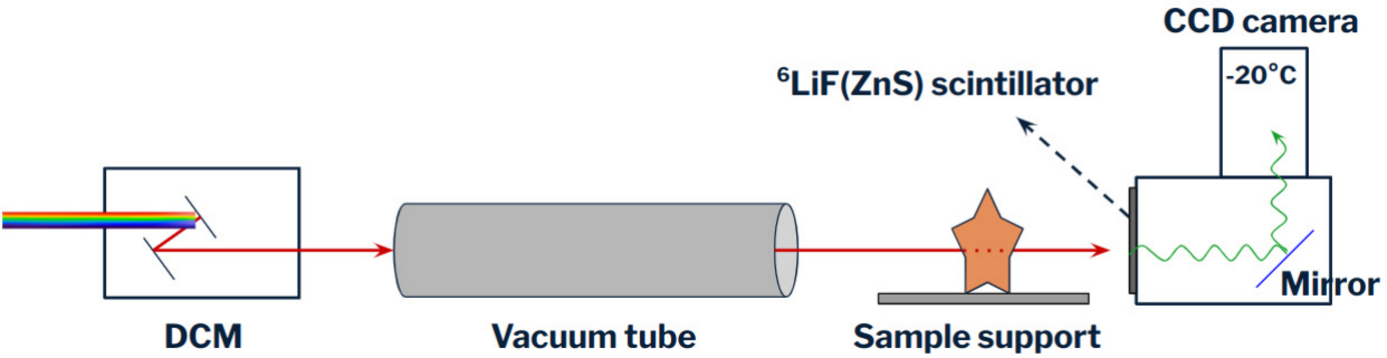
Scheme of the experimental setup used at BOA. With the DCM a monochromatic beam is obtained, which reaches the sample after passing through a vacuum tube; transmitted neutrons hit the scintillator, which produces photons that can be collected by the CCD camera.

**Figure 7:**
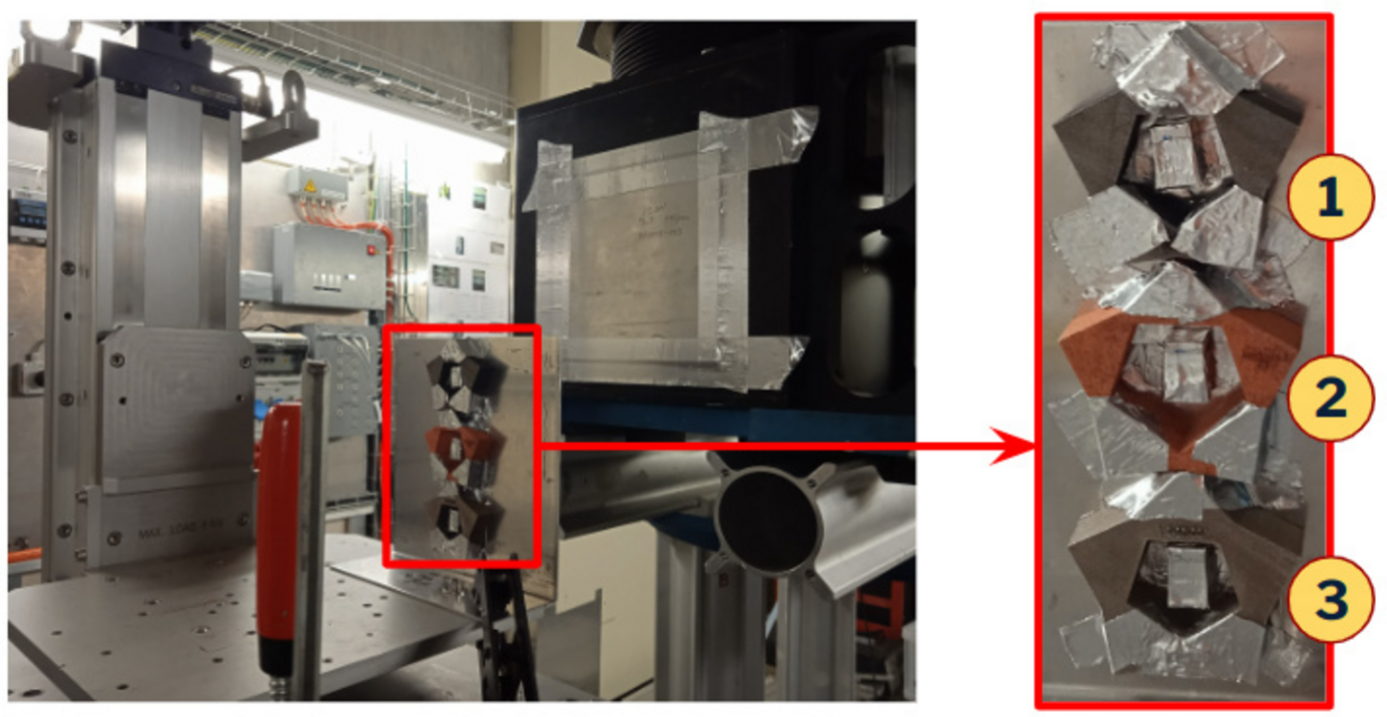
Experimental disposition of the samples: (1) 16MnCr5 low-carbon steel, (2) copper, (3) 316L stainless steel. In the center of each sample is the corresponding powder.

In addition to the DCM and the detector system, the experimental setup consisted of a pin-hole to collimate the beam, a vacuum tube to avoid flux losses and a sample support, a stage with vertical movement placed on one of the available motorized stages. Fig. 6 ▸ shows a scheme of the described setup. (The DCM is arranged horizontally, with the crystal holders rotating vertically; in the figure it is represented vertically only for a better understanding of the scheme.) The three samples were arranged on an aluminium support, fixed with aluminium tape; this plate was then positioned vertically on the sample support with vertical movement. The respective powder used for the production was placed in the center of each sample, contained in a small aluminium box. This configuration is illustrated in Fig. 7 ▸. Aluminium was used because of its very small neutron cross section (NIST, 2021 ▸); no glue was used to avoid scattering interference with the signal from the sample.

#### 2.3.4. Data acquisition

After wavelength selection with the DCM, an open beam image is collected, with no sample in the field of view of the camera. This measurement is used in the analysis to take into account the non-homogeneity of the beam. After that, the stage is raised until the first sample is entirely in the camera field of view, and a radiograph is taken. The vertical movement is repeated until the images are taken for all the samples. Then the next wavelength is set and the procedure restarts.

The stage with the samples is placed close to the scintillator and distant from the DCM exit, so as to have the best possible spatial resolution. In order to increase statistics, five images per sample were acquired for each step, with an integration time of 90 s per image. During data processing, the median of these five radiographs is obtained.

In order to select an appropriate wavelength range, the entire range available at the BOA facility, from 1 to 20 Å, and the expected values of sample *d* spacing have been considered. Thanks to their almost spherical symmetry, the most useful Miller indices to study would be (211) for the body-centered cubic (b.c.c.) structure and (311) for the face-centered cubic (f.c.c.) structure. However, these indices cannot be selected using the DCM setup. Therefore, indices (110) for 16MnCr5 steel (b.c.c.) and (111) and (200) for the other two materials (f.c.c.) have been chosen. Table 3 ▸ shows the expected 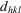 values for the selected Bragg edges. The *d* spacing relative to the copper sample has been calculated using the theoretical value of the lattice parameter (*a* = 3.6147 Å) (Simon *et al.*, 1992 ▸). Since the other two samples are alloys, it is not possible to have a single theoretical value of the lattice parameter, because it depends on the specific concentration of the elements. Therefore, the experimental *d* spacing obtained from the analysis of 16MnCr5 and 316L powders is used to calculate the lattice parameter.

Following these considerations, a wavelength range from 2.5 to 4.4 Å has been chosen, with a step of 0.02 Å.

### 2.4. Calibration

Due to the incident beam divergence, the real wavelength hitting the sample is different from the one set in the DCM (Morgano *et al.*, 2014 ▸); therefore, it is necessary to calibrate the experimental setup. Here a b.c.c. powder was used, in order to compare data of a well known material with theoretical values.

A box containing the b.c.c. powder was placed at the exit of the DCM, so that it covered the entire field of view. For the purpose of studying the difference between experimental and theoretical values of the Bragg edge position, a radiograph was taken, and then the Gaussian fitting method, explained in Section 3, was performed on every single pixel of the image. The (110) edge is considered because it is the most intense.

**Figure 8:**
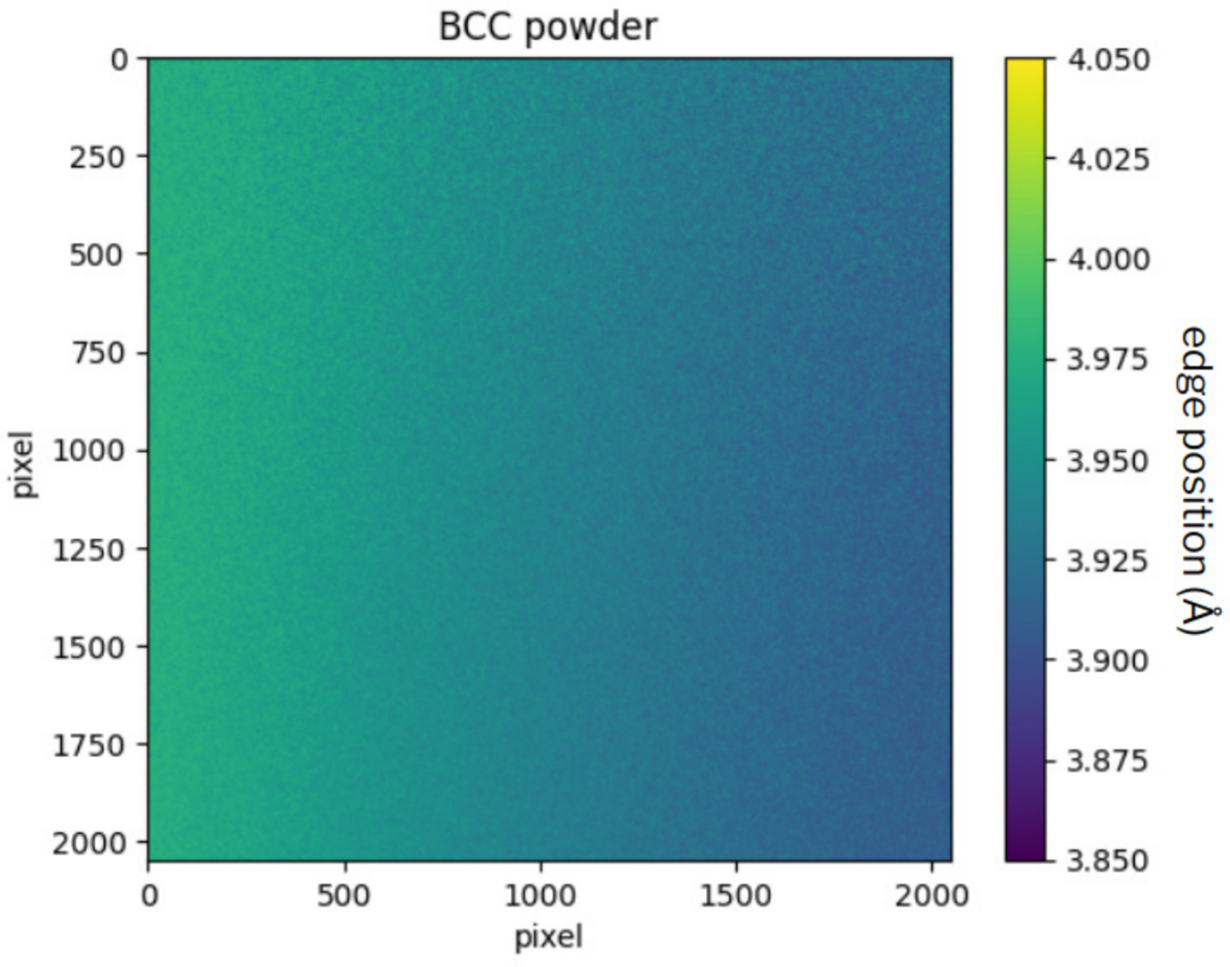
Bragg edge position map, obtained from the Gaussian fitting for each pixel. The color scale ranges from 3.85 to 4.05 Å. The horizontal gradient is evident.

**Figure 9:**
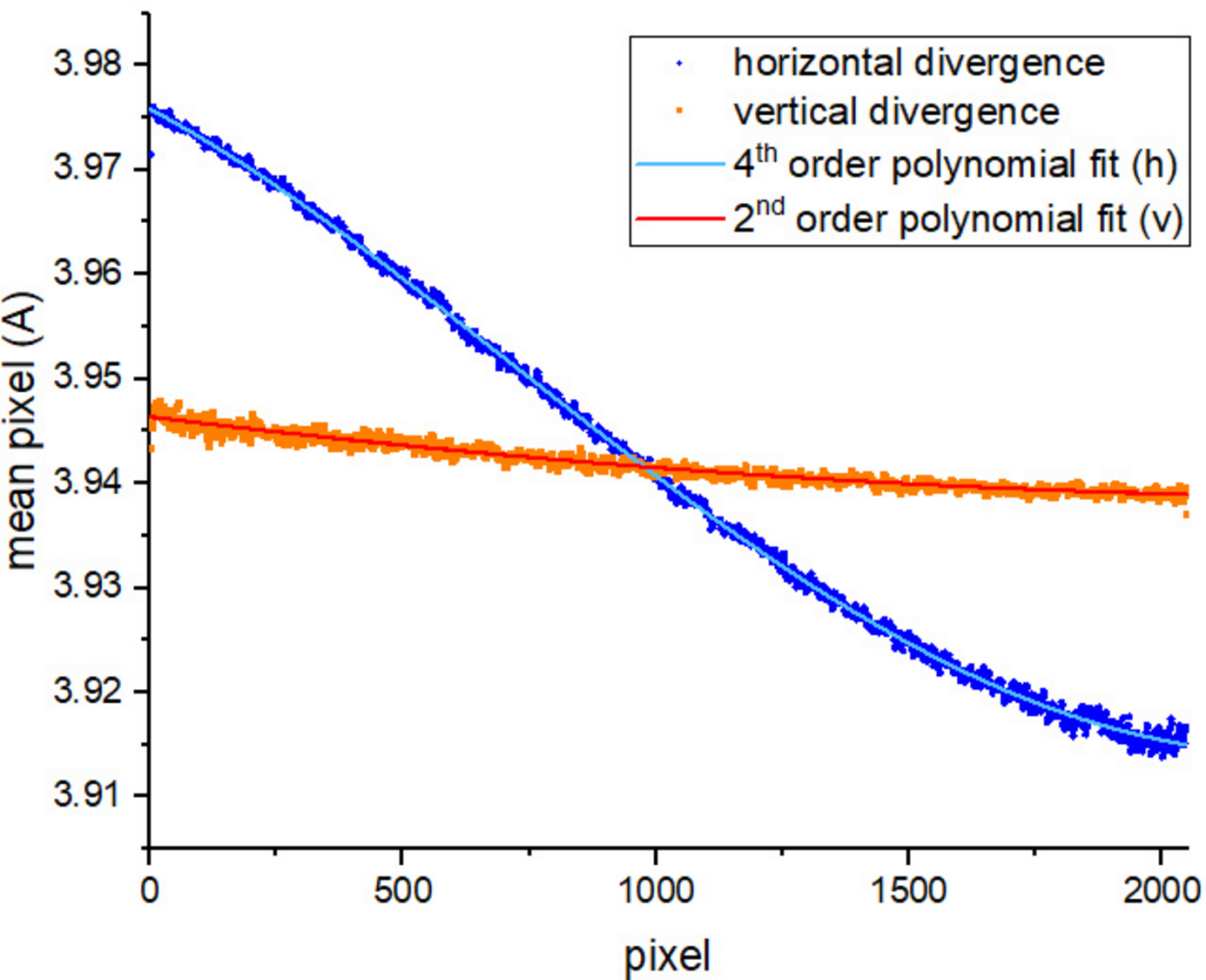
Horizontal and vertical divergence of the edge position map; the horizontal divergence is predominant. A polynomial fitting is performed.

First, this measurement is used to quantify the divergence 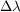. Looking at the edge position map obtained from the fitting process, which is shown in Fig. 8 ▸, the presence of a gradient is evident. In order to evaluate the contribution of horizontal and vertical divergence to the gradient, the mean of every vertical and horizontal array is calculated. These values are then plotted against the pixel number, as shown in Fig. 9 ▸. As can be seen from the plot, the horizontal divergence is dominant, due to the horizontal disposition of the DCM. To describe the divergence trend, a polynomial fitting is per­formed; Table 4 ▸ illustrates the goodness of the fit. From this analysis, it is possible to estimate a value of 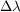 per pixel = (0.288 ± 0.002) × 10^−4^ Å.

To obtain a calibration matrix from the edge position map (Fig. 8 ▸), for each pixel of the image the correction ratio is calculated by dividing the theoretical value of the edge position 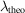 = 4.054 Å (Owen & Yates, 1933 ▸) by the experimental value obtained from the fitting procedure. In this way a correction matrix is obtained, whose pixels have a value equal to 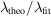. This matrix is multiplied by the edge position maps that are obtained after the Gaussian fitting on the sample images.

Then, from the study of the pure copper powder used to produce one of the samples, another correction factor is obtained. Since this powder covers only a small region of the image, the analysis described above was used to obtain two mean values of the correction factor, one for the (200) edge and the other for the (111) edge. An average of the two correction factors is calculated. Also this mean value is multiplied by all the pixels of the edge position maps that are obtained after the fitting process.

## 3. Results and discussion

**Figure 10:**
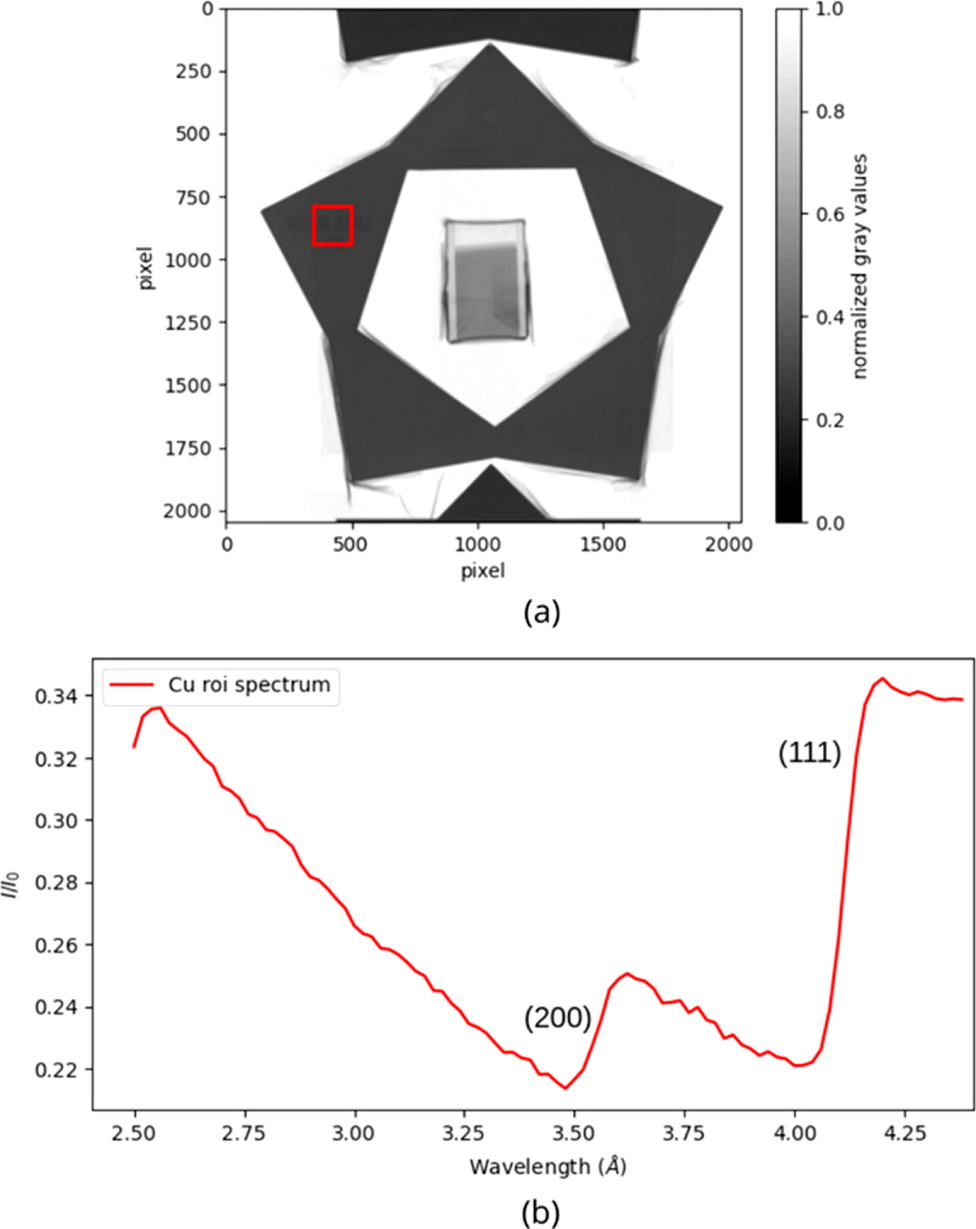
(*a*) Neutron radiography averaged over wavelength for the copper sample; the red rectangle indicates where the transmission spectrum has been obtained. (*b*) Plot of the transmission intensity against wavelength for the copper sample; the Bragg edges (200) and (111) are clearly visible.

The results presented in this work are based on the study of the transmission spectrum, which is the transmitted intensity 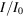 plotted against the wavelength of the incident beam. Fig. 10 ▸ shows this spectrum for the copper sample.

**Figure 11:**
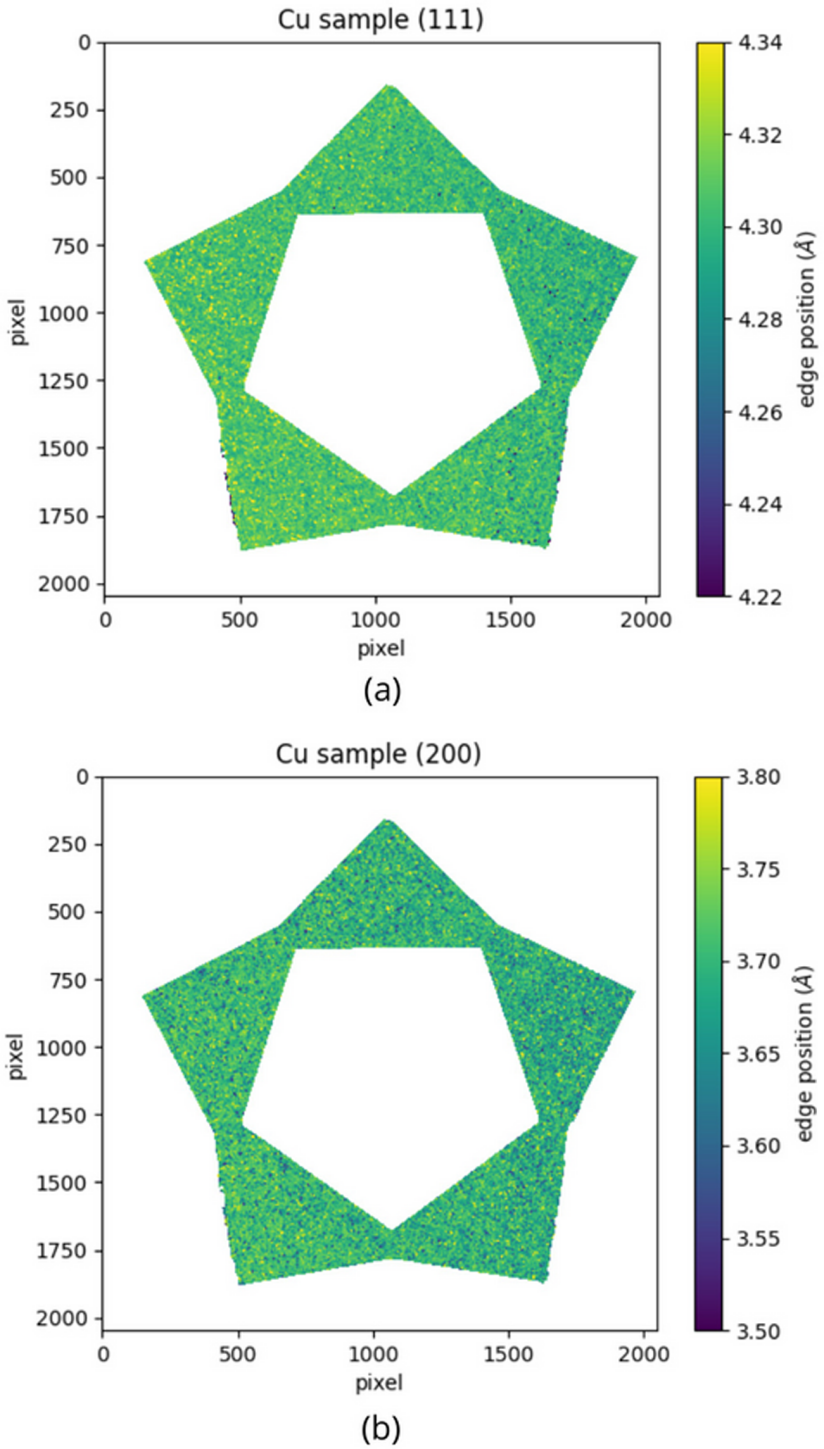
Bragg edge position maps of the copper sample. (*a*) Gaussian fitting for the (111) edge; the color scale ranges from 4.22 to 4.34 Å. (*b*) Gaussian fitting for the (200) edge; the color scale ranges from 3.50 to 3.80 Å.

In order to characterize the Bragg edges and to determine the wavelength at which they occur, a Gaussian fitting method has been adopted. This method consists of taking the derivative of the edge, obtaining a Gaussian-like peak. A subsequent fit allows for the correlation of the Gaussian mean and width with the specific Bragg edge parameters. The parameters that are of particular interest for this analysis are the position and the width of the Bragg edge. To conduct this analysis a Python script developed by Busi *et al.* (2021 ▸) was used. The Gaussian fitting has been performed for each pixel of the sample images, studying the (111) and (200) edges for the copper and the 316L samples, and the (110) edge for the 16MnCr5 sample, as explained in Section 2.3.4. In Fig. 11 ▸ the Bragg edge position maps of the copper sample are shown, both the (111) (*a*) and the (200) (*b*) edges. The uncertainty on the edge position is ∼10^−3^ Å. These maps are homogeneous over the entire surface. The information yielded by these maps is the basis for the characterization of the crystalline structure of the materials under study.

Before presenting the results, a key methodological aspect regarding the experimental geometry must be addressed. The samples were oriented with the build direction parallel to the incident neutron beam; as a consequence, each measurement represents an integrated average along this axis. In this configuration, localized edge effects are not spatially resolvable, as they are superimposed on the bulk signal. Nevertheless, the Bragg edge broadening provides significant insights into the average lattice strain trends within the bulk. While this specific orientation limits the spatial resolution of localized edge effects, the BENT technique remains highly effective in characterizing the overall structural state and global strain trends of the material.

### 3.1. Elastic lattice strain

At the Bragg edge position, the Bragg condition for a given set of planes 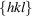 [equation (1)] simplifies to
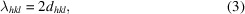
where the *d* spacing 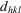 is the distance between crystalline planes of a given family (*hkl*). Starting from the edge position maps it is hence possible to obtain *d*-spacing maps, which allow, through the elastic lattice strain 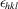, a comparison with the theoretical value 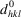 (Busi *et al.*, 2021 ▸): 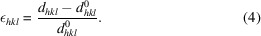
Therefore 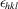 gives information on how much the crystallographic structure can be affected by mechanical or thermal processes carried out on the samples.

The *d*-spacing value is calculated for each pixel of the sample and the powder, and then, according to equation (4), the elastic lattice strain is obtained. The copper sample is made of pure copper, so it is possible to calculate a theoretical value of the *d* spacing 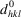, thus obtaining both the strain for the sample and the compositional discrepancy for the powder. This double comparison allows one to study the effects of the manufacturing process, and also to confirm the absence of strain in the powder. However, for the other two samples it is not possible to calculate a theoretical 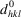, as described in Section 2.3.4, and therefore the mean *d*-spacing value of the powder region is taken as a reference.

**Figure 12:**
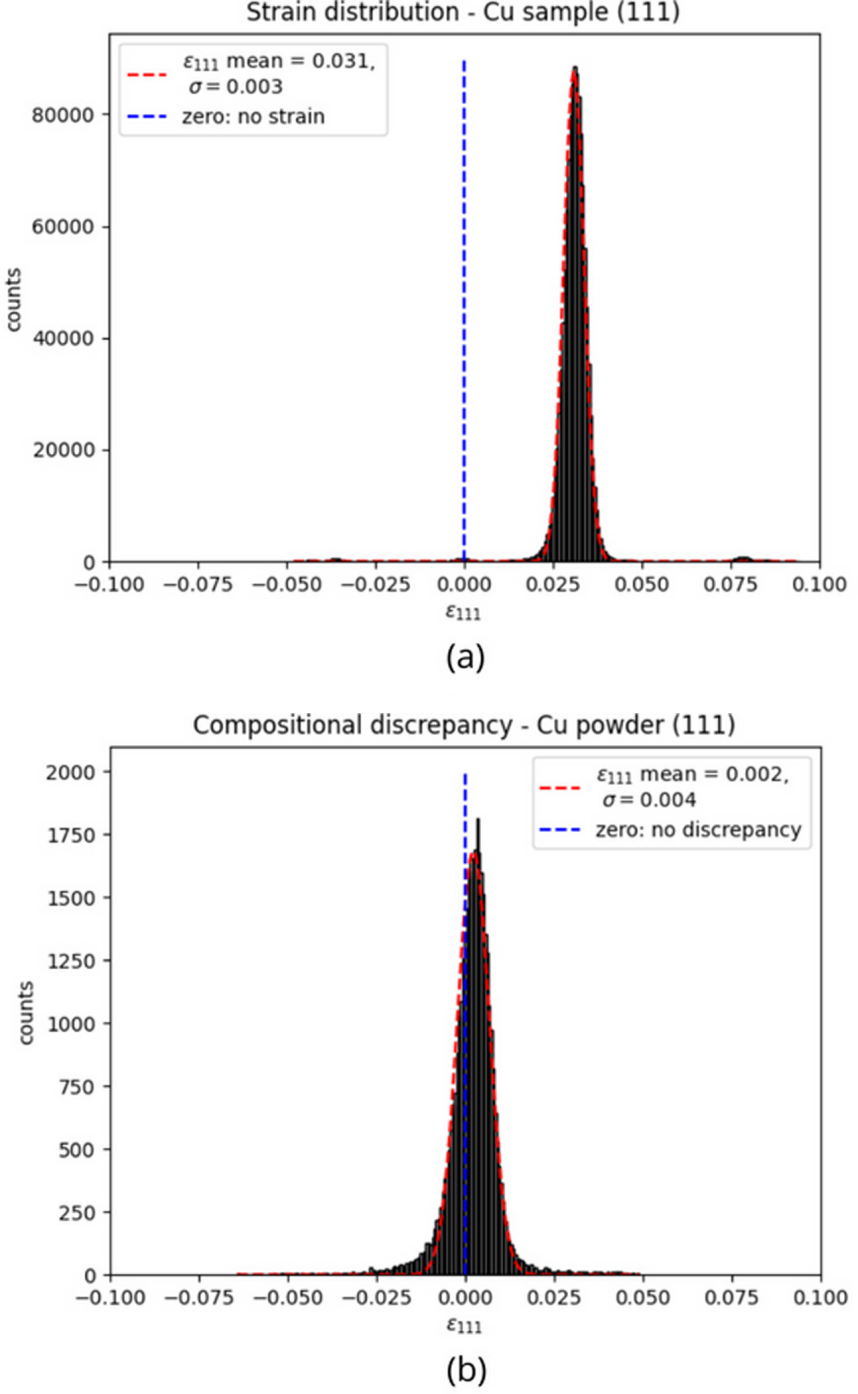
Elastic lattice strain distribution for the copper sample (*a*) and compositional discrepancy distribution for the copper powder (*b*), relative to the (111) Bragg edge. A Gaussian fitting is performed. The mean value of the powder distribution is compatible with zero at a 5% significance level.

The 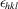 distributions relative to the copper sample (*a*) and powder (*b*) are shown in Fig. 12 ▸. A fit with a Gaussian function was performed; the parameters obtained from the fitting are summarized in Table 5 ▸. As expected, the mean value of the elastic lattice strain distribution for the powder region is compatible with zero at a 5% significance level, while the sample distribution is not centered at zero. This indicates that the PBF-LB manufacturing process started from a copper powder without strain and produced a sample with an average elastic lattice strain of (0.031 ± 0.003).

**Figure 13:**
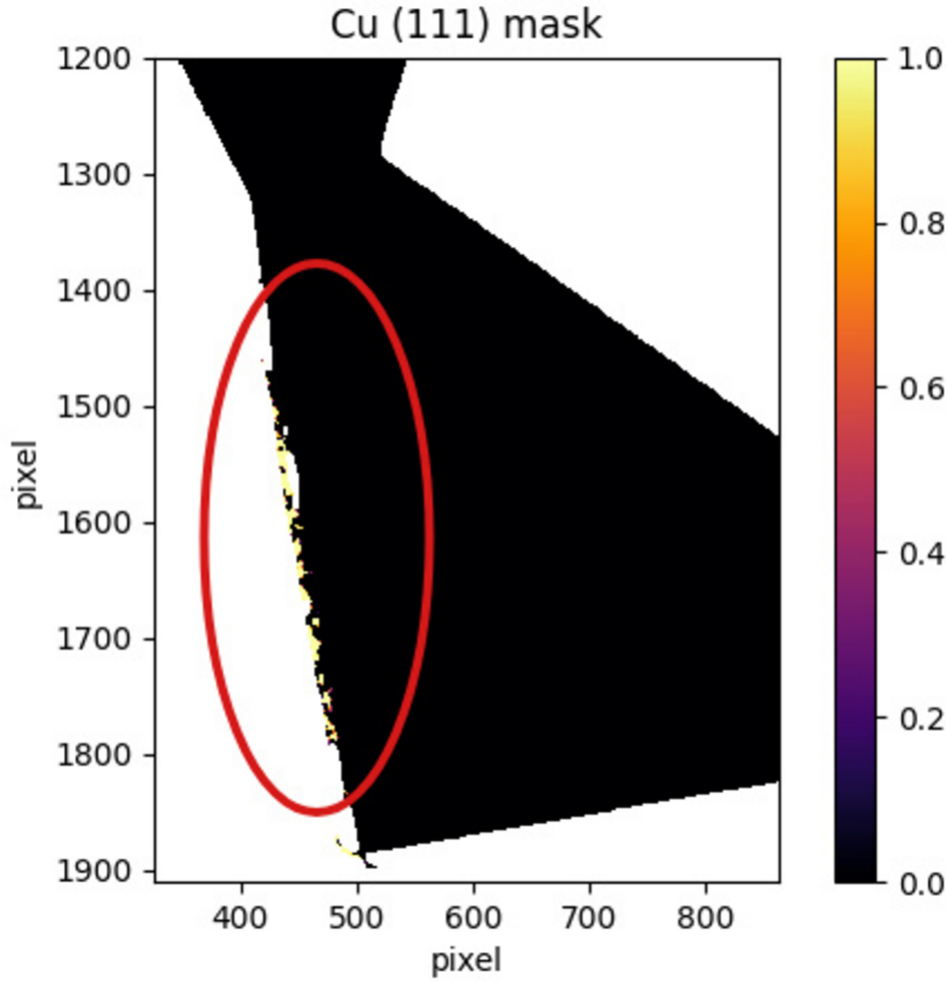
Zoom on the bottom left tip of the copper sample. A mask is applied on the (111) elastic lattice strain map: yellow pixels correspond to the ones that contribute to the small peak around 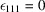. These pixels are mainly located at the sample edge, where the aluminium tape was positioned during the experimental measurements. The red line indicates the area with the highest density of searched pixels.

The elastic lattice strain distribution of the sample exhibits a small peak around 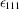
 = 0. A mask has been applied to the strain map in order to identify which pixels contribute to this structure. Fig. 13 ▸ highlights pixels with 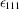 around zero, within an area chosen as an example. These pixels are mainly located at the edge of the sample, where the aluminium tape was used in the experimental setup. The presence of this small peak in the strain distribution could therefore be due to this experimental effect.

The same analysis was carried out for the (200) Bragg edge of copper. Also in this case the powder compositional discrepancy (−0.001 ± 0.009) is compatible with zero as expected, while the sample strain (0.026 ± 0.007) is greater and not compatible with zero.

As mentioned above, for the other two samples the elastic lattice strain is evaluated using the average *d* spacing of the relative powder as 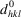. Table 6 ▸ summarizes the results obtained from the analysis. All samples exhibit positive strain values. Furthermore, the observed consistency across different materials suggests that residual strains induced by the AM process are primarily governed by production parameters rather than material properties.

### 3.2. Density of crystallographic defects

**Figure 14:**
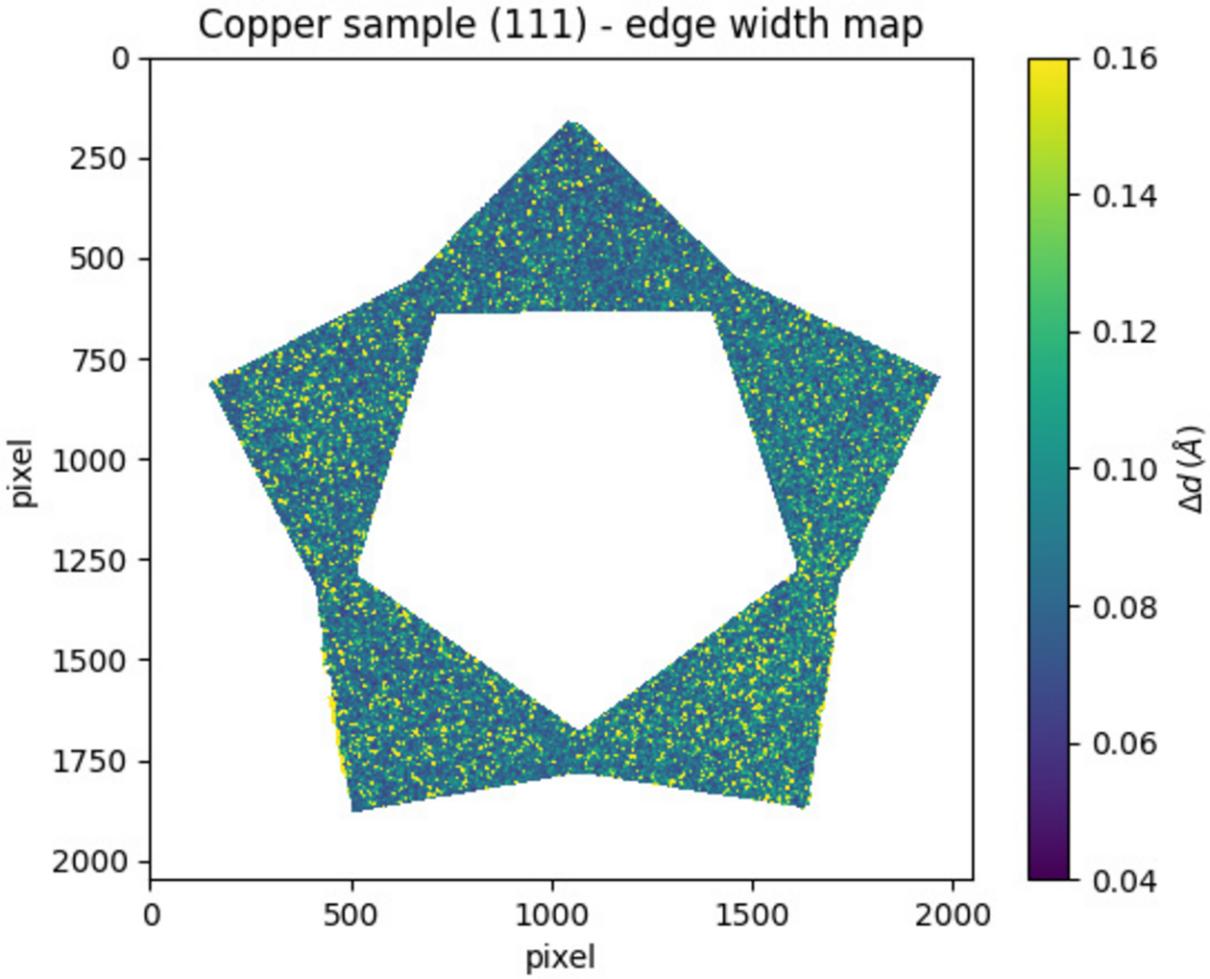
Width map for the (111) Bragg edge of the copper sample. The color scale ranges from 0.04 to 0.16 Å.

**Figure 15:**
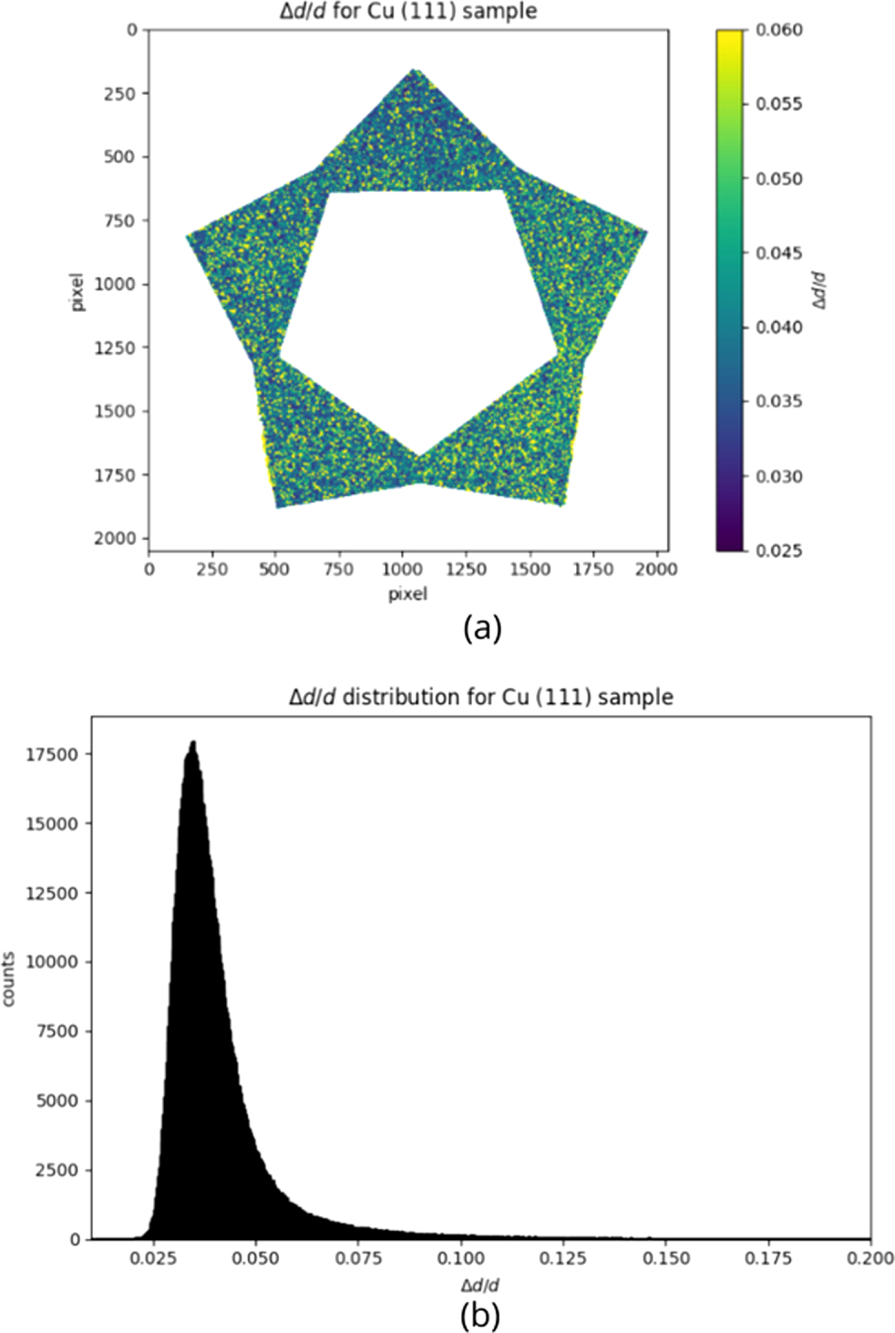
Copper sample, (111) Bragg edge. (*a*) 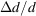 map; the color scale ranges from 0.025 to 0.060. The map is not homogeneous, but presents higher values of 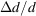 mainly in the right and bottom right tips of the star. (*b*) 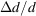 distribution; the peak is around 0.034 and it is not symmetric: the right wider tail reflects the higher values highlighted in the map.

The lattice parameter of an alloy may exhibit spatial variations across the object, resulting in a broadening of the Bragg edge (Sato *et al.*, 2015 ▸). Fig. 14 ▸ shows the (111) Bragg edge width map for the copper sample. To study this effect, the ratio 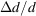 is evaluated, where 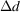 is the width of the Bragg edge and *d* is the *d* spacing. According to the typology of the crystal structure of the materials, the following hierarchy is expected: 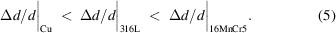
Fig. 15 ▸ shows the 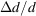 map (*a*) and distribution (*b*) for the (111) Bragg edge of the copper sample. The map is not homogeneous: the values of the pixels are higher in some areas than in others. These pixels are the yellow ones on the map, and they contribute to the wider right tail of the distribution. Since the pixels with higher values are not located on horizontal or vertical bands, the effect is not due to the DCM or other experimental conditions, but it could reflect real characteristics of the sample deriving from the production process. This hypothesis is supported by the fact that the other two samples also present areas with pixels at higher values, but located in different zones. Table 7 ▸ summarizes the characteristics of the 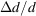 distribution of the three samples. The hierarchy between different materials is slightly different from the expected one [see equation (5)]. The hypothesized hierarchy is based on the crystalline structure of the materials and on the effect of the intrinsic impurities present in the alloy. The fact that the experimental 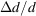 results do not reflect this hierarchy indicates that the component of defects induced by the manufacturing process is dominant over the intrinsic impurities of these structures.

As regards the comparison between different Bragg edges of the same material, the 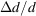 value is not expected to be the same for different indices (*hkl*), because some directions are more sensitive than others. Nevertheless the difference between the (111) and (200) edges of the steel 316L sample is very large; this could be due to the strong texture present in this sample, as described in Section 3.3.

### 3.3. Texture

In a polycrystalline material, texture occurs when the crystallographic axes of the grains follow some preferred orientation, and the percentage of crystals having such a preferred orientation determines the degree of texture (Kocks *et al.*, 2000 ▸). The texture can be induced in a sample by a mechanical process or thermal gradient, for example during production processes (Busi *et al.*, 2021 ▸). Material properties such as strength, deformation behavior or resistance to radiation damage can be highly dependent on the material’s texture and related changes in microstructure. Therefore, understanding the texture that occurs after a specific process is fundamental in order to qualify the production process itself (Malamud *et al.*, 2014 ▸).

**Figure 16:**
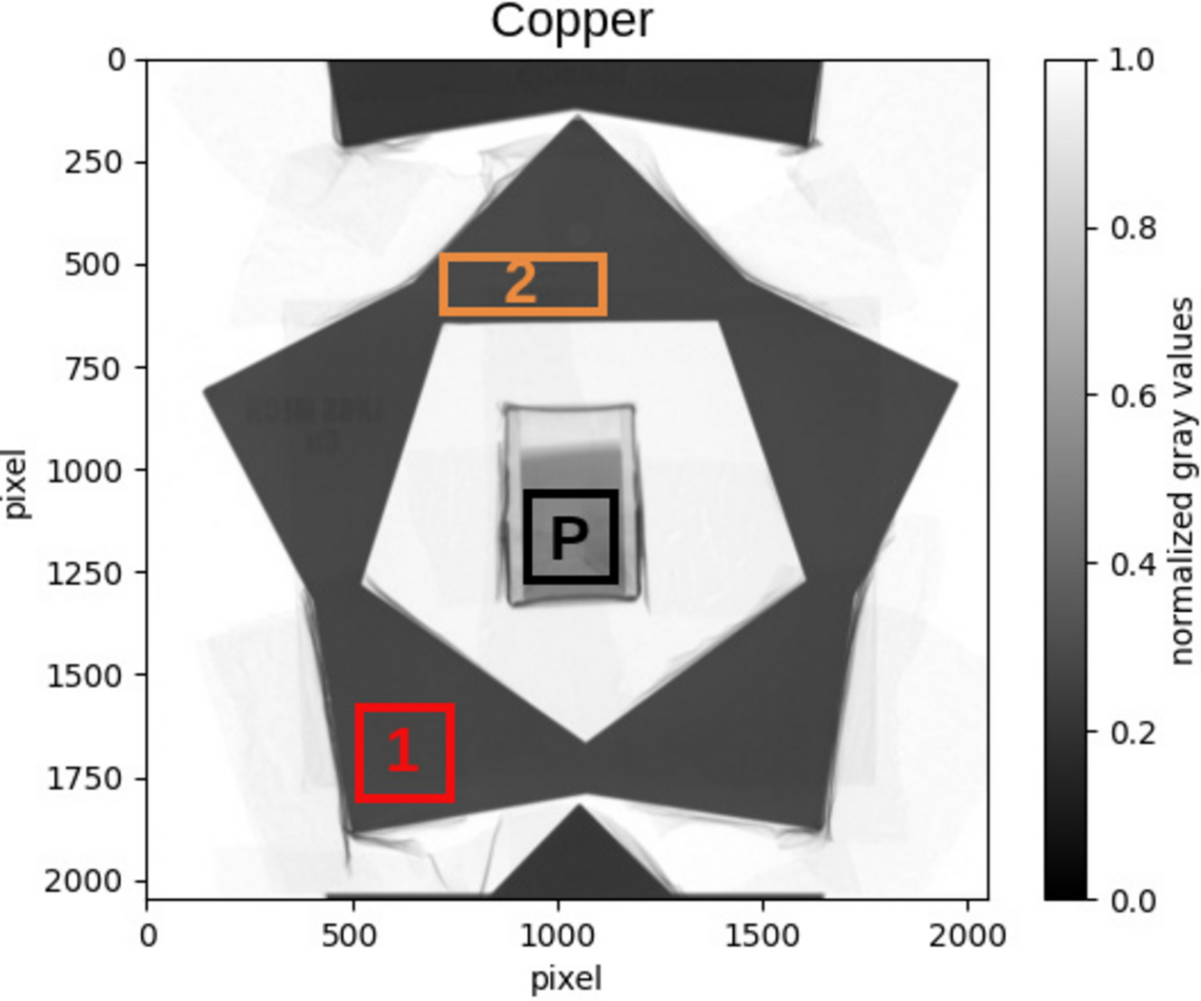
Different regions for which the transmission spectrum is here reported. The Cu sample is shown as an example, but the same selection has been done for each dataset.

Here a qualitative study of the effects of texture on the Bragg edge pattern is presented. Since the texture is expected to be only in the sample and not in the powder, this analysis was carried out by comparing the transmission spectrum of the sample with that of the reference powder. In the sample area the transmission spectrum is studied for different regions. Since the spectrum is similar in all the different regions considered, here for greater clarity only the spectrum relating to two areas, shown in Fig. 16 ▸, is reported.

**Figure 17:**
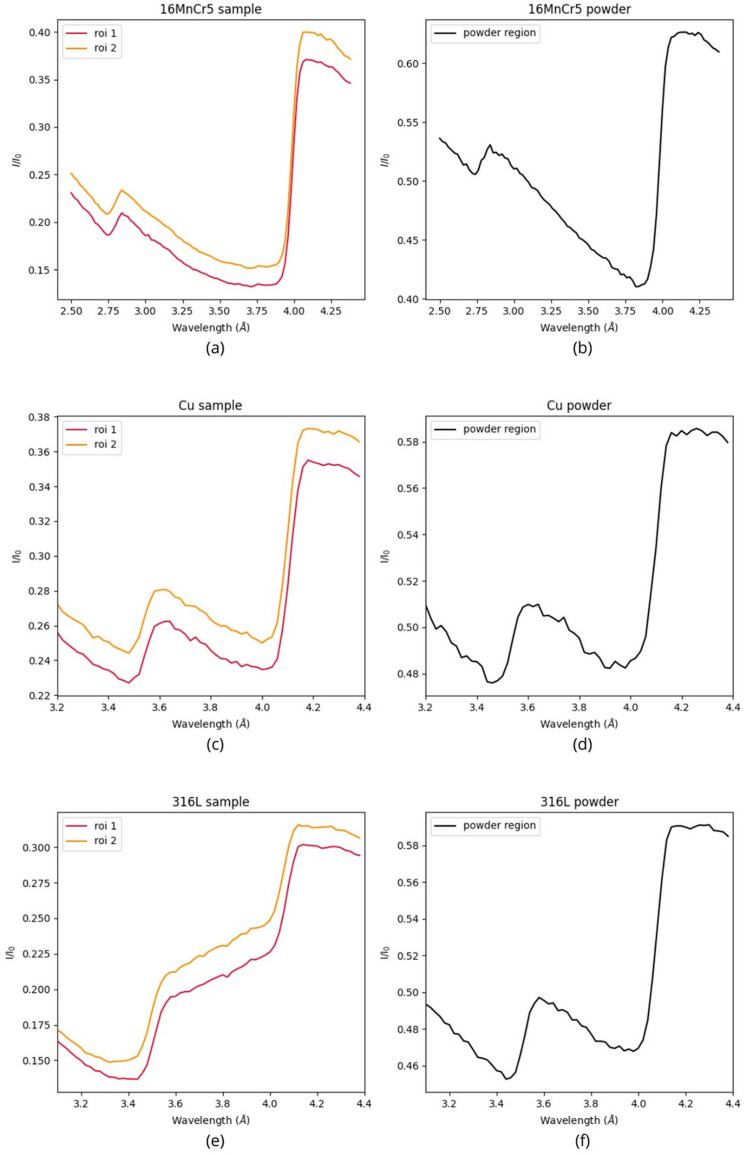
Sample (*a*), (*c*), (*e*) and powder (*b*), (*d*), (*f*) transmission spectra for the three materials. For improved presentation, the orange spectrum has been shifted vertically by 0.02. Due to the different thicknesses of the sample and powder, for clarity the transmission reported on the *y* axis has a different scale in the two cases. The regions between two different Bragg edges have a different slope in the sample and in the powder. This effect is very pronounced in the case of the austenitic steel 316L (*e*), (*f*).

In Fig. 17 ▸ the transmission spectra for the three samples and the related powders are shown. The spectra corresponding to the two different regions have been vertically shifted by 0.02 relative to each other, to make the plot more readable. There are some differences between the transmission spectra of the sample and the powder spectra; in particular, the most relevant difference is in the shape of the region of the spectrum between two consecutive Bragg edges. This region should be a straight line like in the theoretical reference spectrum, but in the sample it becomes a concavity or a convexity. A greater variation of this region compared with the expected behavior corresponds to a greater degree of texture. Among the three samples, the austenitic steel 316L [Figs. 17 ▸(*e*) and 17 ▸(*f*)] has the highest degree of texture, while the least difference compared with theory occurs in the copper spectrum [Figs. 17 ▸(*c*) and 17 ▸(*d*)]. The stronger crystallographic texture in 316L austenitic steel is primarily attributed to its low thermal conductivity (∼15 Wm^−1^ K^−1^). In materials with low conductivity, the heat generated by the laser during the manufacturing process remains localized at the upper layer, establishing a thermal gradient along the build direction. This condition promotes epitaxial grain growth across multiple layers, whereas in high-conductivity materials like copper (∼401 Wm^−1^ K^−1^) rapid and multi-directional heat dissipation leads to a more isotropic microstructure (Herzog *et al.*, 2016 ▸; Thijs *et al.*, 2013 ▸; DebRoy *et al.*, 2018 ▸). The low-carbon steel 16MnCr5 exhibits an intermediate degree of texture compared with the other two samples, consistent with its thermal conductivity (∼41 Wm^−1^ K^−1^).

## 4. Conclusions

Bragg edge neutron imaging has been applied to the AM field, characterizing three innovative star-shaped metal samples, produced with PBF-LB technology. This preliminary analysis aims to investigate the effects of the production process on the manufactured object. In order to obtain their position and width, the (110) Bragg edge of a 16MnCr5 low-carbon steel sample, the (111) and (200) edges of a copper sample, and the (111) and (200) edges of a 316L stainless steel sample have been analyzed using a Gaussian fitting method. The elastic lattice strain, the density of crystallographic defects and the texture of the three samples have been studied. Each of these quantities allowed us to highlight the presence of differences between the crystalline structure of the samples and the reference powders. The observed consistency in strain values across different materials suggests that the residual stresses induced by the manufacturing process are primarily determined by the production conditions rather than the type of material. The non-homogeneous distribution of the density of crystallographic defects further indicates that AM-induced imperfections are dominant over intrinsic material impurities. Furthermore, qualitative analysis of the Bragg edge patterns revealed that the austenitic steel 316L, due to its low thermal conductivity, exhibits the most pronounced crystallographic texture among the investigated samples. Therefore, this analysis confirms that the manufacturing process affects the crystalline structure of the metal, varying the distance between crystalline planes and introducing different degrees of anisotropy. The introduction of this type of defect can affect the overall functionality of the manufactured part; therefore it is very important to correlate them with the parameters of the production process. To further extend this study, advanced characterization techniques – specifically neutron diffraction and neutron tomography – will be employed to achieve a deeper understanding of the internal structure of the samples. In addition, future research will involve the production of samples with varying manufacturing parameters to systematically investigate their direct influence on the crystallographic structure.

## Figures and Tables

**Table 1:** Main dimensions of the sample based on material density

Material	ρ (g cm^−3^)	*h* (mm)	*l*_1_ (mm)	*l*_2_ (mm)	*d*_1_ (mm)	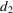 (mm)
316L	8.0	18.0	7.1	19.4	63.0	45.0
16MnCr5	7.8	18.0	7.1	19.4	63.0	47.0
Cu	8.9	18.0	4.9	16.5	63.0	47.0

**Table 2:** PBF-LB process parameters for each metal material

Parameter	316L	16MnCr5	Cu
*P* (W)	175	175	175
*S* (mm s^−1^)	1450	1150	300
*H* (µm)	70	70	70
*L* (µm)	40	40	30
VED (J mm^−3^)	43.1	54.3	277.8
ρ (%)	99.6	99.9	90.5

**Table 3:** Expected values for the *d* spacing 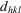 and the lattice parameter *a*

	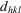 (Å)	*a* (Å)
16MnCr5 (110)	2.031 ± 0.07	2.872 ± 0.010

Cu (111)	2.0869	3.6147
Cu (200)	1.8074	

316L (111)	2.08 ± 0.01	3.59 ± 0.03
316L (200)	1.79 ± 0.02	

**Table 4:** Polynomial fitting of the horizontal and vertical divergence

	Horizontal	Vertical
Order of polynomial fit	4th	2nd
Residual sum of squares	5.117 × 10^−4^	4.211 × 10^−4^
*R* ^2^	0.99932	0.95824

**Table 5:** Elastic lattice strain fit parameters for the Bragg edge (111) of the Cu sample and powder, obtained from the fitting with a Gaussian function

	Sample	Powder
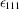	0.031046 ± 0.000006	0.0023 ± 0.0002
FWHM	0.00680 ± 0.00002	0.0105 ± 0.0005

**Table 6:** Elastic lattice strain values for the three samples

16MnCr5 (110)	0.027 ± 0.002
Cu (111)	0.031 ± 0.003
Cu (200)	0.026 ± 0.007
316L (111)	0.028 ± 0.005
316L (200)	0.031 ± 0.005

**Table 7:** Density of crystallographic defect distribution for the three samples (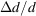 values)

16MnCr5 (110)	0.039
Cu (111)	0.034
Cu (200)	0.034
316L (111)	0.032
316L (200)	0.041

## Data Availability

Data supporting the results reported in this article are stored on the PSI servers and can be accessed upon request.
